# Assessing the representational structure of softness activated by words

**DOI:** 10.1038/s41598-023-35169-6

**Published:** 2023-06-02

**Authors:** Müge Cavdan, Nedim Goktepe, Knut Drewing, Katja Doerschner

**Affiliations:** 1 Experimental Psychology, Justus-Liebig-Universität Gießen, 35390 Gießen, Germany; 2 Philipps-University Marburg, 35037 Marburg, Germany; 3 National Magnetic Resonance Research Center, Ankara, 06800 Turkey

**Keywords:** Psychology, Human behaviour

## Abstract

Softness is an important material property that can be judged directly, by interacting with an object, but also indirectly, by simply looking at an image of a material. The latter is likely possible by filling in relevant multisensory information from prior experiences with soft materials. Such experiences are thought to lead to associations that make up our representations about perceptual softness. Here, we investigate the structure of this representational space when activated by words, and compare it to haptic and visual perceptual spaces that we obtained in earlier work. To this end, we performed an online study where people rated different sensory aspects of soft materials, presented as written names. We compared the results with the previous studies where identical ratings were made on the basis of visual and haptic information. Correlation and Procrustes analyses show that, overall, the representational spaces of verbally presented materials were similar to those obtained from haptic and visual experiments. However, a classifier analysis showed that verbal representations could better be predicted from those obtained from visual than from haptic experiments. In a second study we rule out that these larger discrepancies in representations between verbal and haptic conditions could be due to difficulties in material identification in haptic experiments. We discuss the results with respect to the recent idea that at perceived softness is a multidimensional construct.

## Introduction

Material qualities of objects influence how we interact with them, we tend to indent soft things, we grasp fragile things gently, and apply somewhat more force when a slippery surface starts to slide from our hands. To learn about materials, we usually explore them with our hands, while watching, hearing, and smelling the results of this exploration. Through this, associations between visual and haptic (and also olfactory and auditory) perception are established, and this knowledge is important for guiding and shaping future interactions^1–10^. These associations are so strong that even when direct visual or haptic sensory information is not available, we are able to make statements about typical (or expected) material properties^11^. For instance, seeing a brand name of a hand cream may instantaneously elicit vivid expectations about the cream’s viscosity, stickiness, smell, translucency, and color. In fact, there are many examples in research demonstrating the existence of strong multisensory associations. For example, Adams et al.^12^ showed that the glossiness of a surface can be used as a proxy for judging its slipperiness. Similarly, Paulun et al.^13^ showed that the viscosity of liquids can be inferred from static images. In our own recent work, we showed that softness dimensions (viscosity, granularity, and surface softness^14^) that were derived from haptic rating experiments can similarly be found from the ratings of visually perceived materials in static (close-up images) and dynamic scenes (videos showing the manual exploration of materials)^14^. This high correspondence between static visual and haptic information suggests that, although the materials in the study were primarily defined through their mechanical and tactile properties, this information was also indirectly accessible through visual shape and texture cues alone. In that study, we reasoned that participants likely relied on their prior knowledge about the materials when rating tactile and mechanical properties, that is, the provided visual information activated the representation of a specific material, which allowed the participants to ‘fill in’ or ‘read out’ the ‘missing’ sensory information^14^.

A representation consists of a multitude of associations, between sensory impressions, but likely also includes conceptual knowledge^15,16^ and may contain links to episodic memory. The representation of a material is not the material itself and therefore ‘storage’ of the most relevant information might involve some level of abstraction. As a consequence, drawing on a representation can also alter our perception. The memory color effect is one striking example where the representation of a typical object color (e.g., bananas are yellow) modulates color appearance^17–19^. Similarly, Metzger and Drewing^20^ found a memory softness effect: If participants expected to probe the compliance of a harder material (e.g., a tennis ball) the same stimulus is perceived to be harder as compared to when they believe to probe a softer material (e.g., a sponge). Alley et al.^1^ showed expectations based on prior knowledge about the typical kinematic properties of a material modulated how material properties were perceived.

Softness is an important mechanical property of materials that is primarily perceived through touch^21–29^, yet, we^14^ and others^30–36^ have found that also by visual inspection alone, humans make reasonable judgements about primary haptic qualities of objects—most likely by activating the relevant material representation. Here, we want to directly investigate the nature of this representation when it is are activated by words only, i.e., the name of a material. In a previous study^14^ we showed, when participants judge softness-related qualities of materials, there are similarities as well as differences between visually and haptically perceived softness. Here we ask how the representational structure of softness activated by words compares to the ones activated by visual and haptic exploration. The degree of overlap with one or the other may allow us to draw conclusions about what type of sensory information is predominantly associated with a specific material quality or material category.

**Figure 1:**
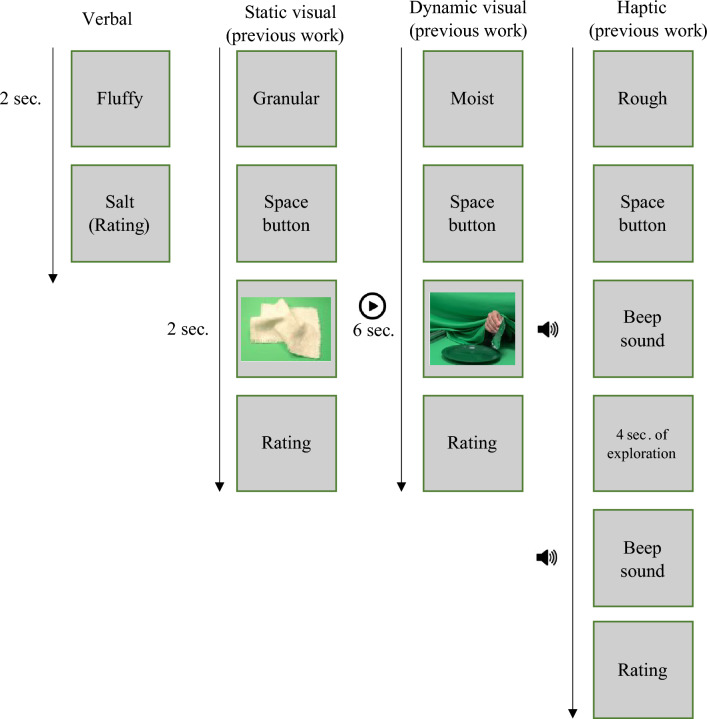
Time course of a trial across conditions in Study 1. In all conditions, an adjective to be rated was presented on the screen first. After pressing a button, in the verbal condition, the name of a material was presented on the screen for 2 s; in the static visual condition (previous experiment^3,14^): a static image presented for 2 s; in the dynamic visual condition (previous experiment^3,14^): a 6 s movie clip showing a manual exploration was presented; and in the haptic condition (previous experiment^3,14^): a beep sound signaled the start of 4 s of exploration, and another beep signaled the end of the trial. In all conditions, participants indicated how much a given adjective applies to the material on 5-point Likert type scale.

We probed this representational space by presenting written material names, a paradigm that has been shown in object perception to trigger, for example, the same representations as visual depictions^37,38^. For every material name, in an online study, 132 participants rated the material’s properties based on sensory adjectives (see Fig. 1). The same materials and adjectives were also used in our previous work^3,14^. Using a Principal Component Analysis (PCA) we determined the dimensionality of the verbally-derived perceptual softness space and compared it to those derived from our earlier haptic and visual experiments^3,14^. Results showed that the verbally-derived softness space is a multidimensional construct with similar dimensions as haptic and visual softness. Using linear discriminant classification we next tested whether material ratings based on words (i.e., material names) can be used to correctly classify material ratings obtained in the previous haptic and visual experiments^3,14^. Overall, we found that the representation of perceived softness obtained from the verbal study is similar to those obtained from previous perceptual experiments. However, it is more similar to that obtained from the visual experiment. In order to find out whether this outcome might be related to a difference in identifiability in visual and haptic experiments, we conducted a second study where participants either visually (static images) or haptically explored a (real) material, then identified the material (freely naming it) and subsequently assigned it to the best fitting softness dimension (i.e., surface softness: fluffy/hairy/velvety, viscosity: wobbly/moist/sticky, granularity: granular/sandy/powdery, deformability: deformable/elastic/inflexible) or control (roughness: rough/smooth). Despite an overall low performance in the naming task in both conditions, recognition of materials was somewhat better in the visual condition. However, despite these differences, materials in both conditions were assigned to the same softness dimensions. Consequently, the difference in material identifiability in the two conditions did not account for the differences in visual and haptic softness, and by extension, does not explain why verbal and visual representations were more similar to each other than verbal and haptic representations.

## Results

### Perceptual softness space derived from material names

Our first aim was to determine the structure and dimensionality of the softness space derived from the verbal condition and to compare it to those derived from earlier haptic and visual experiments. To this end, we first assessed interparticipant consistency of individual ratings of 19 materials (presented as words, see Table 1 and methods for trial details) using 15 adjectives to check to what extent participants responded similarly to the individual material names. Sufficiently high similarity in responses would allow us to use average material-adjective ratings across participants in the PCA. Bartlett scores of the PCA, which indicate the degree to which each material is associated with the extracted dimensions, were used in Procrustes analyses to measure the similarity between the perceptual softness space derived from words with those derived from previous visual and haptic experiments. Since the Procrustes analysis yielded a high agreement between conditions (verbal [this study], visual -static and dynamic—and haptic [previous work^3,14^]), we submitted the average responses from each condition to a combined PCA in order to assess the more fine-grained structural differences between conditions. Numerical results of these analyses are reported next.

**Table 1:**
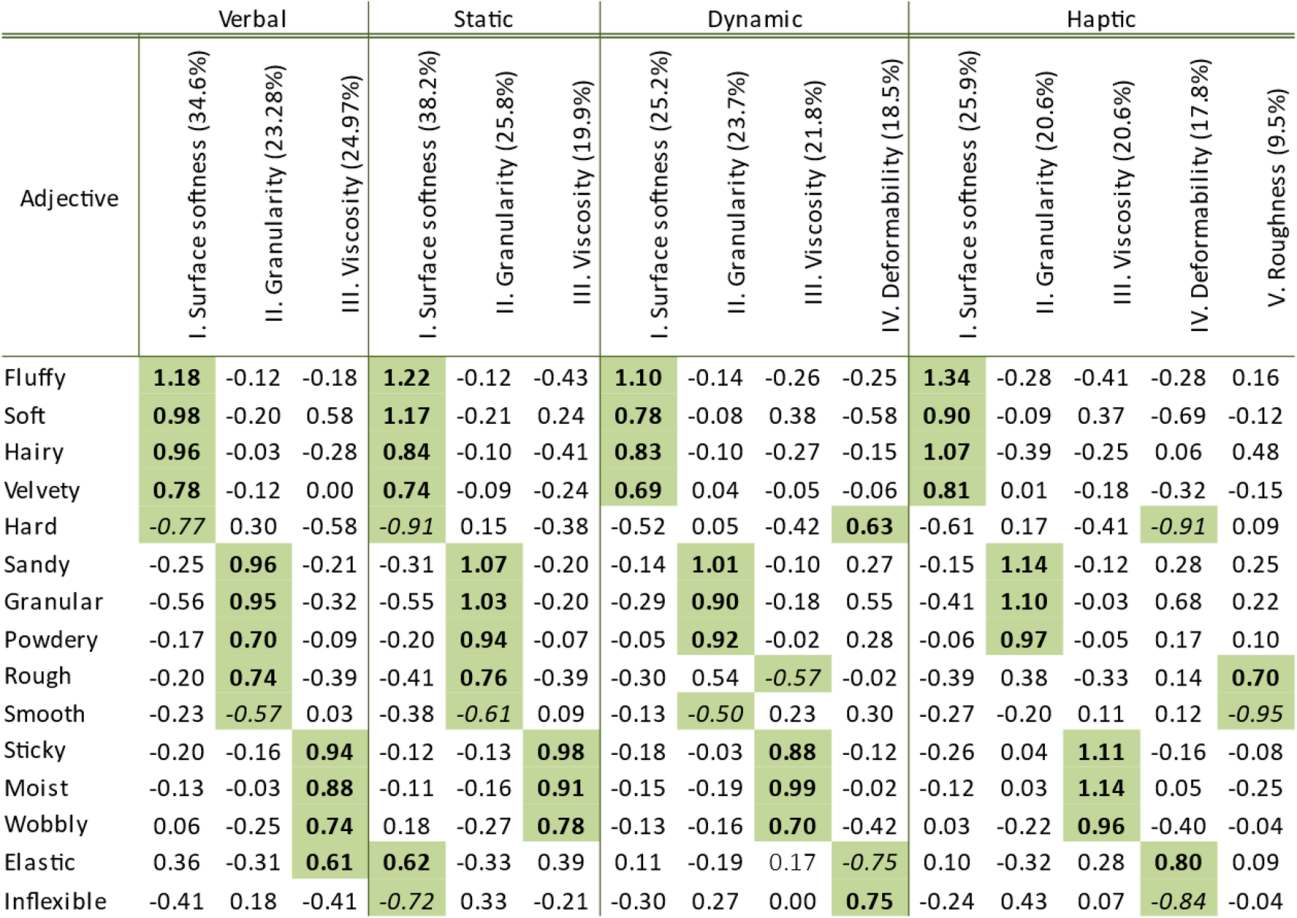
Rotated adjective loadings for verbal, static visual, dynamic visual, and haptic conditions.

Colors indicate high adjective loads (> 40% of mean variance per adjective explained, which corresponds to loads of 0.64 verbal, 0.68 static visual, 0.62 dynamic visual, 0.74 haptic), or that an adjective loads higher on a specific factor than on others. Bold if the loading is positive, italic if the loading is negative^14^.

#### Consistency

Interparticipant Pearson correlation coefficients revealed that only one of the participants was not consistent with all other participants (*r*_*mean*_ = 0.20). We excluded this person from further analyses. Pearson correlations between the remaining (131) participants were significant (*p* < 0.001), ranging between 0.30 and 0.82 (*r*_mean_ = 0.59), and were therefore, comparable to those in earlier studies^3,14^. Despite a potential proneness of verbal information to variability in individual interpretations, the high correlations suggest that participants rated the properties of the presented materials in a similar way. Given this, we averaged the rating data across participants and proceeded with the PCA.

#### Dimensionality

To determine the dimensionality of perceptual softness derived from material names we submitted the averaged ratings for the different materials to a covariance based principal component analysis (PCA). The Keyser-Meyer-Olkin (KMO) value was 0.53 and the Bartlett test of sphericity was significant, χ^2^ (105) = 344.70, *p* < 0.01, which suggests that it was appropriate to conduct a PCA. Principal components were extracted based on the Kaiser-criterion and rotated using the varimax method. Three extracted rotated components explained 82.87% of the total variance. The first component which we called *surface softness* (high adjective loadings from: *fluffy*, *hairy*, *velvety*, *soft*, and *hard*) accounted for 34.6% of the variance. The second component *viscosity* (adjective loadings: *wobbly*, *sticky*, *moist*, and *elastic*) accounted for 24.97% of the variance. Finally, the third component *granularity* (adjective loadings: *sandy*, *powdery*, *granular*, *rough*, and *smooth*) accounted for 23.28% of the variance.

Values in Table 1, which lists the rotated adjective loadings for the verbal study (Study 1) along with those, obtained from previous visual and haptic experiments, indicate that overall, the extracted components and loading patterns were similar to those obtained in our previous haptic and visual softness studies (see Cavdan et al.^14^). There we found that *surface softness*, *granularity*, and *viscosity* were common to haptic & static and dynamic visual conditions and explained most of the variance in ratings, whereas *deformability* only appeared in the dynamic visual and haptic conditions, and roughness was only specific to the haptic condition^14^. However, we also see that the adjective loadings obtained from Study 1 (verbal) appear to be somewhat more similar to those obtained from the static visual condition^14^.

In order to quantitatively assess the structural similarities between the three components (surface softness, granularity, and viscosity) that are common to all conditions (verbal, visual, haptic), we performed a Procrustes analysis on the Bartlett scores of materials. A Bartlett value is the score that shows the loading of a material in each dimension (i.e., hand cream loading score per *surface softness*, *granularity, viscosity, deformability* and *roughness*). From this analysis we calculated the sum of squared errors that remains after mapping between any two softness spaces.

Overall, the error between conditions was low (verbal and static visual: 0.12, verbal and dynamic visual: 0.32, verbal and haptic: 0.33), indicating a good fit between the derived softness spaces^39^. We used a bootstrapping approach^40^ for significance testing. First, for every space comparison, we created 10,000 pseudo Bartlett values by shuffling the respective empirical Bartlett values. Then, we calculated the Procrustes error for each empirical and pseudo comparisons. All empirical mapping errors were significantly lower than chance as they were within the first 2.5 percentile of the pseudo errors (equivalent of two-tail significance test with α = 0.05).

### Combining perceptual softness spaces from verbal, visual and haptic experiments

After confirming significant similarity between verbally, haptically, and visually derived softness spaces we next conducted a combined PCA, in order to determine more fine-grained structural differences. Mean ratings from all four conditions (verbal, static visual, dynamic visual, haptic) were submitted to a single PCA. The KMO value was 0.71 and Bartlett’s test of sphericity was significant, χ^2^ (105) = 1550.73, *p* < 0.01 suggesting that PCA was suitable for the averaged rating data across four conditions. We extracted the principle components for the combined data based on Kaiser-criterion and rotated them using the varimax method. Four components accounted for 87.98% of the total variance (see Table 2). The first component, labelled *surface softness,* accounted for 30.50% of the variance. Adjectives loading high on this component were *fluffy*, *velvety*, *soft*, *hairy*, and *hard*. The second component, labelled *granularity,* accounted for the 26.52% of the variance. Here, the adjectives *powdery*, *sandy*, *granular*, *inflexible*, and *elastic showed high loading*. On the third component, which accounted for 22.25% of the variance, adjectives *sticky*, *moist*, and *wobbly* loaded highly and was thus labelled *viscosity*. Finally, the component labelled *roughness* explained 8.71% of the variance. On this component only the adjective *rough* and *smooth loaded highly.*

**Table 2:**
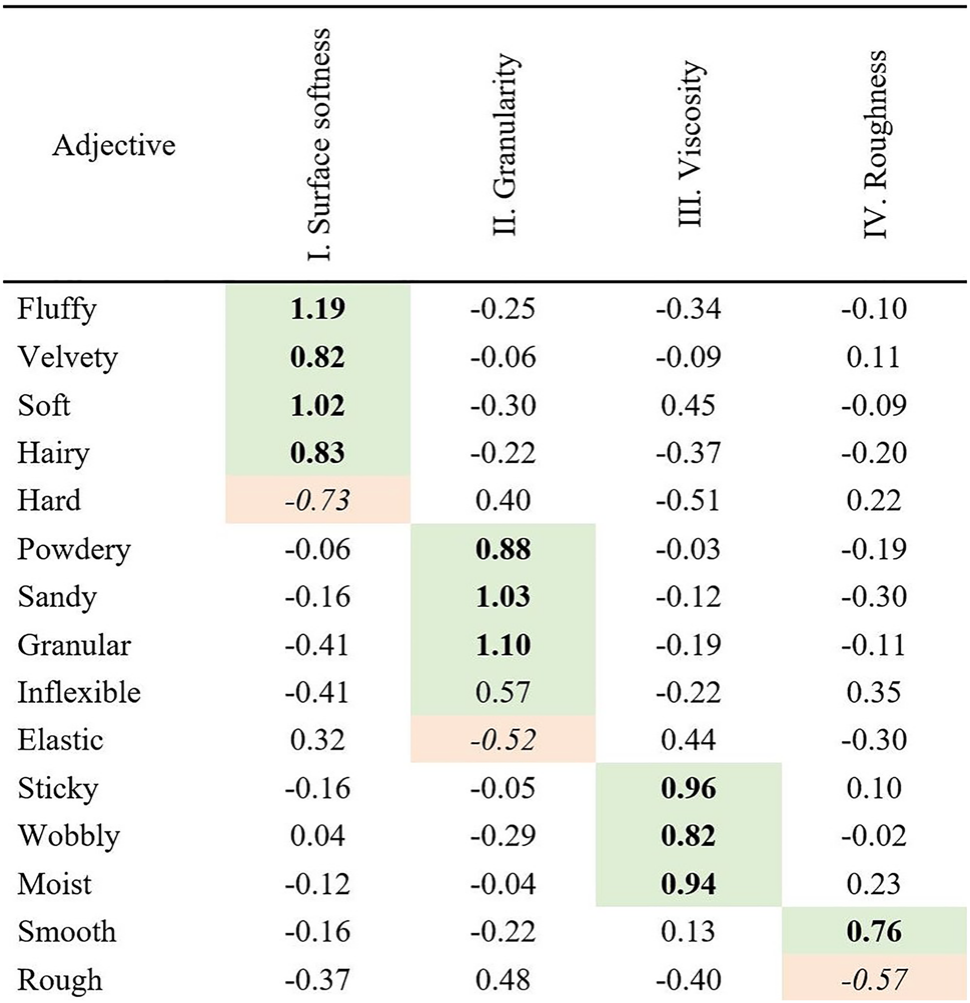
Rotated adjective loadings from the combined PCA analysis.

Components determined based on high adjective loadings (> 40% of the mean variance corresponding to loading 0.66 or highest on a specific dimension). Bold if loading is positive and italic if loading is negative.

Now we could proceed with the correlation analyses that allowed us to directly test the similarity between the verbal condition and remaining three. Bartlett values of each condition were calculated from the combined PCA. The scores that are obtained from each condition are correlated with the scores from the verbal study (i.e., verbal-haptic, verbal-static, verbal-dynamic).

**Figure 2:**
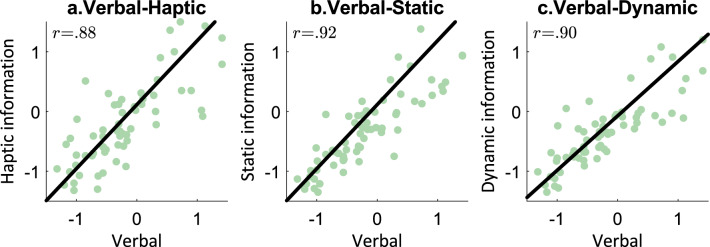
Correspondence between perceptual softness spaces derived from the verbal and other conditions,—x-axes values show Bartlett scores of materials obtained from the verbal condition while y-axes indicate Bartlett values derived from haptic, static visual, and dynamic visual conditions: (**a**) verbal-haptic, (**b**) verbal-static visual, (**c**) verbal-dynamic visual. Each datapoint represents a rotated Bartlett score of a material in each extracted perceptual softness dimension (see Supplementary Fig. 3).

All correlations were significant at *p* < 0.001 level (Bonferroni-corrected for three tests). Figure 2 shows the correlations between the extracted Bartlett scores of the softness spaces derived from the verbal study and those obtained in the previous work: verbal-haptic (*r* = 0.88), verbal-static (*r* = 0.92), and verbal-dynamic (*r* = 0.90). These strong correlations indicate high similarity between verbally derived and perception-based perceptual spaces.

### Predicting perceived material softness in visual and haptic experiments from material name responses

Next, we tested to what extend it is possible to predict perceived material surface softness, granularity, or viscosity from a verbally activated representation of a material. To this end we trained classifiers (six-fold validation) on adjective ratings of materials that loaded high in one of the three softness dimensions (see Supplementary Fig. 3) for each condition (static visual, dynamic visual, haptic) to predict responses in the verbal condition and vice versa. Specifically, we used the ratings from the following materials; surface softness: velvet, fur, and cotton balls; granularity: salt and sand; viscosity: hand cream and hair gel.

**Figure 3:**
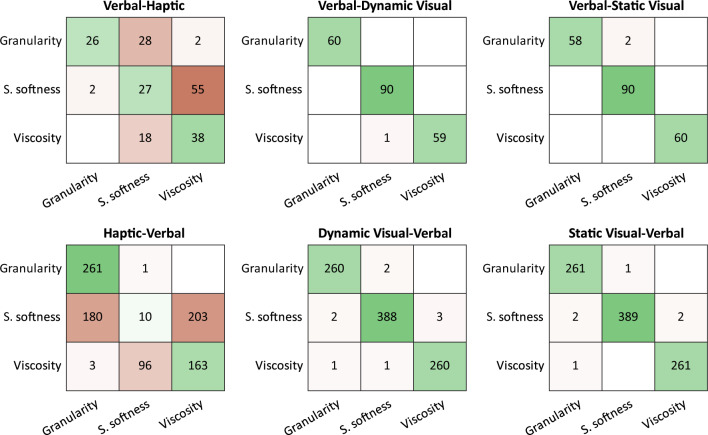
Confusion matrices showing softness dimension classification performance across conditions. Linear discriminant classifiers were trained on adjective ratings in Verbal, Static-Visual, Dynamic-Visual, and Haptic conditions. Overall material classification performance was as follows: Verbal classifier: Haptic: 46.43%, Dynamic: 99.52%, Static: 99.05%. Haptic classifier: Verbal: 46.24%, Dynamic Visual classifier: Verbal: 99.02%, Static Visual classifier: Verbal: 99.35%. x-axes show the predicted dimension while y-axes show the true (as for that condition) dimension.

Results in Fig. 3 show, that overall, classifiers performed better than chance (chance level = 100/3 = 33.3), and that classifiers trained on visual and verbal conditions predicted each other nearly perfectly (all accuracies > 99%).

In contrast, prediction of the haptic condition from the visual or verbal conditions and vice versa was substantially lower (~ 45% accuracy, see Fig. 3). Specifically, the classifier trained on haptic data frequently confused *surface softness* with *granularity* and *viscosity*, and confused *viscosity* with *surface softness* when classifying the softness dimensions of materials based on their ratings from the verbal (Fig. 3), and visual conditions (Supplementary Fig. 1).

**Figure 4:**
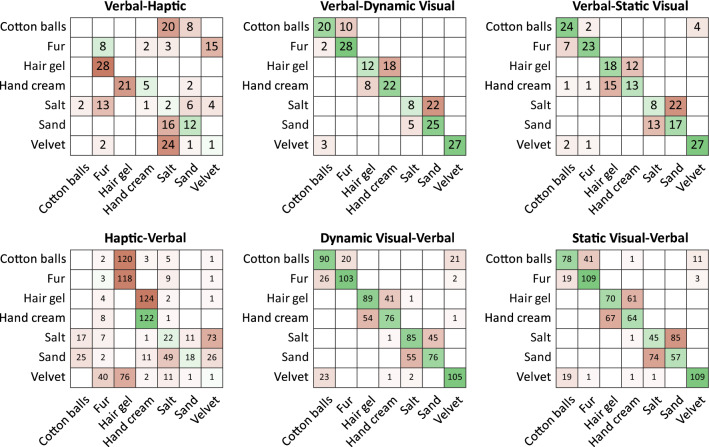
Confusion matrices showing the material classification performance of Verbal, Haptic, Visual Static, and Visual Dynamic linear discriminant classifiers trained on material ratings in the respective conditions. Material classification performances as follows. Verbal classifier: Dynamic: 67.62%, Haptic: 14.29%, Static: 61.9%. Haptic classifier: Verbal: 18.1%. Dynamic classifier: Verbal: 68.05%. Static classifier: Verbal: 58.02%. Numbers in each row add up to number of materials representing each dimension × number of participants in each classified condition.

The high structural similarity of softness spaces shown above might explain why seeing a hair gel commercial, or reading the name of a hair gel product we are familiar with, so vividly evokes a sense of how the gel would feel when touched. A correct mapping, however, between would require high correspondence between the actual representations of a hair gel’s soft qualities evoked by words, pictures, and touch, and if true, it should be possible to classify a material based on its softness properties in one domain also in another domain. To test this hypothesis, we next, trained a second set of linear discriminant classifiers for each condition (see Fig. 4), again using the adjective ratings of materials that are highly loaded to one softness dimension to directly classify materials in other conditions (six-fold validation). The classifiers trained on verbal, static visual, and dynamic visual data were able to make cross-condition material classifications better than chance (Chance level = 100/7 = 14.29%). Although significant, the cross-classification performance of the classifier trained on haptic may be negligible as it could reliably classify only *hand cream* in other conditions. Other classifiers were not able to classify materials based on haptic qualities. The classification errors were primarily due to confusing materials of the same softness dimension (e.g., hair gel and hand cream, are both *viscous* materials).

### Identification and classification of soft materials

**Figure 5:**
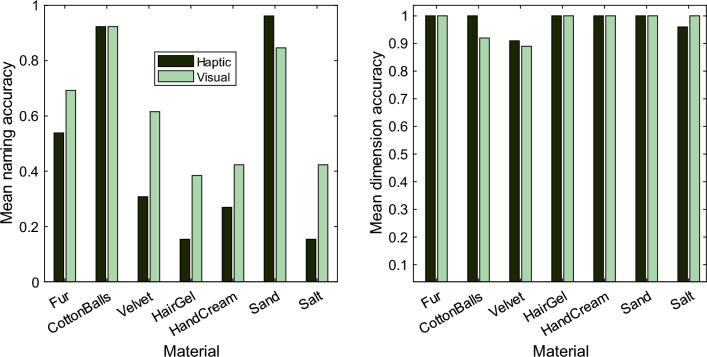
Mean accuracy of the naming (left) and dimension classification task (right) of the Study 2, for each material in the visual (darker bars) and the haptic (lighter bars) conditions (*N* = 52).

It is possible that the larger structural differences between softness spaces derived from verbal and haptic conditions that we observed in Procrustes and classification analyses above, are in fact due to the fact that in the haptic condition it might have been more difficult to identify/categorize materials compared visual conditions. To test this hypothesis we investigated in a second study how identifiable materials are when presented visually and haptically, and how well participants could assign a given material into one of five softness dimensions (surface softness, viscosity, granularity, deformability, roughness), each represented through specific adjective groups (1, Fluffy/hairy/velvety; 2, Wobbly/moist/sticky; 3, Granular/sandy/powdery; 4, Deformable/elastic/inflexible; 5, Rough/smooth). Participants’ responses from the naming task were inspected and translated from German into English. Participants often used multiple words to define an object, and sufficiently similar terms (e.g., velvet/velvet cloth, cream/hand cream/sun cream, sand/fine sand, fake fur/fur) were accepted as valid responses. Responses labelled as invalid can be found in Supplementary Figs. 4–6. From the valid responses, we next calculated the proportion of accurate responses for each material, across participants, and separately for visual and haptic conditions (see Fig. 5, left). In general, naming performance in visual and haptic conditions was rather low (mean accuracy, 0.62 (*SD* = 0.20) and 0.47 (*SD* = 0.32), respectively), with the exception of two materials: cotton balls, and sand. These were also the two only materials for which identification performance in the haptic condition was equal or superior to that in the visual condition. For the remaining materials identification was somewhat superior in the visual condition, and an undirected, paired t-test yielded a small but significant overall difference in mean accuracy between the two conditions *t*(6) = 2.49, *p* = 0.047 (Fig. 5, left). While visual identification is statistically superior, it is only marginally so and far from perfect.

We next checked, to what extend stimuli were assigned correctly to a specific softness dimension (1–5), and find that for both modalities (vision and haptic), this assignment was nearly perfect, with no significant differences between modalities (*t* (6) = 0.58, *p* = 0.585). This suggests, that knowing the name of a material is not a prerequisite for activating the ‘correct’ softness representation^3^ (i.e., following the same representation found in the PCA analysis).

**Figure 6:**
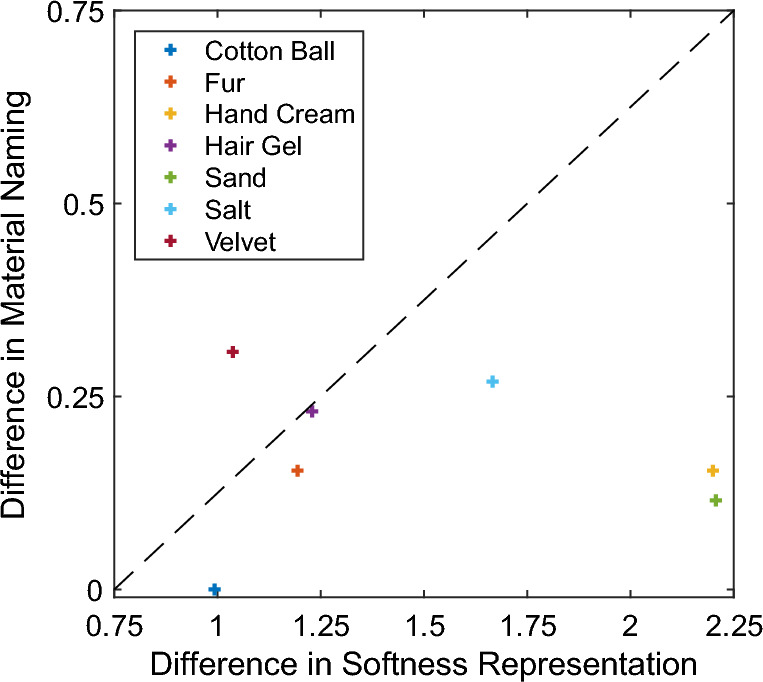
Differences in naming (Study 2) as a function of rating differences between visual and haptic soft conditions. Each point represents these differences for a given material. The dashed line represents the trendline. For both coordinates (x,y) we computed the absolute difference. The difference between visual and haptic softness representation was calculated by calculating the Euclidian distance of each material’s ratings (each, a 15-dimensional point) between visual and haptic conditions.

Finally, to test whether identification differences between visual and haptic modalities are in any way related to differences in the corresponding representational structure of softness, we calculated the Euclidian distance of each material’s ratings (each, a 15-dimensional point) between visual and haptic conditions (data from previous experiment^14^) and correlated these differences with the corresponding differences in identification accuracy (Fig. 6). This yielded a non-significant *r* = − 0.069, *p* = 0.88, and suggests that differences in material identifiability across visual and haptic domains do not relate to differences in the corresponding softness representations.

## Discussion

The perception and categorization of a material is not only determined by just-experienced sensory information, but also by our prior experiences with the material and our general knowledge about it. Repeated interactions with objects and materials lead to the formation of multisensory associations that make up the representational structure of a given material (wood) or material quality (e.g., softness). These representations are thought to not only contain perceptual knowledge, including knowledge about how materials react to physical forces^1,41^, but contain also conceptual knowledge^15^, and entail perhaps even specific episodes of encounter/interaction with a material (remembering how it felt when touching a piece of rough lumber) maybe as much part of the representation as abstract conceptual knowledge (wood is hard and also rough if not sanded down). The associations or connections that make up the structure of a representation allow us to ‘fill in’ information about a material that is not directly available from the stimulus (e.g. see Schmid and Doerschner^42^, for concrete examples see^14^ or^30–36^). Here, we wanted to probe the nature of this representation for soft materials, by activating it through words only.

In previous work we showed that perceived softness is a multidimensional construct^3,14,43^ and we found a similar dimensionality of the softness space when estimated from participant ratings after they haptically explored a material or while they watched static or moving images of a material^14^. In the studies reported here, we wanted to know whether we would find the same nuanced differentiation between different kinds of softness when participants just think of/imagine a specific material (category), as when they felt or saw a material. Indeed, we found that the verbally activated representation of soft materials is also a multidimensional construct, that is highly similar to that obtained from visual and haptic conditions^30,39,44,45^. Specifically, three dimensions were common to verbally, visually, and haptically activated representations: surface softness, granularity, and viscosity.

However, correlations and Procrustes analysis also revealed that the verbally activated softness representation is slightly more similar to that derived from visual conditions, than that estimated from haptic experiments. And following this up we find that specific softness dimensions as well as specific materials are much better predictable from softness judgments between verbal and visual conditions than between verbal and haptic ones (see also Supplementary 1). These asymmetries are quite surprising, given that softness can haptically be directly estimated from the physical properties of materials, while visually and verbally (prompted) judgements of softness are associative^11^. And therefore, one would expect the haptically activated softness representation to be some kind of gold standard, and therefore to be maximally similar to any of the other conditions, since those other (verbal, visual) spaces should be derived from it. Conversely, it could be argued that the visually and verbally derived softness spaces should be more similar to each other since both depend on some filling in by activating the corresponding associative network, i.e., the representation(s). Finally, it could be argued that ratings in the visual and verbal condition were more similar because participants could simply not recognize the materials in the haptic condition, and were therefore perhaps not able to access the correct material representation, which in turn leads to larger discrepancies between softness representations obtained from haptic and other conditions. We directly tested this possibility in the second experiment where people identified materials and assigned them into one of the four dimensions of softness and roughness (control), either visually or haptically. While participants were indeed somewhat better at naming materials in the visual- than in the haptic condition, errors in the haptic condition were in fact limited to confusions of a material with another material of the same softness dimension (e.g., salt confused with sand, see Supplementary Fig. 4). Consequently, despite these naming differences, people assigned materials in both, visual and haptic conditions, nearly perfectly to one of the five softness dimensions^3,14^. This suggests that overall, the correct naming of a material is not prerequisite for activating the ‘correct’ softness representation. While identifiability may account for the finer within dimension confusions, it appears not to shape how softness spaces estimated from haptic and visual experiments are overall organized. The overall high structural correspondence of softness spaces activated by words and by touch might provide a direct account of how haptic information might be ‘filled in’ when direct sensory information is lacking. Future studies could investigate multisensory ‘filling in’ in more detail by comparing softness judgments of familiar and novel materials under ambiguous or impoverished sensory conditions.

Participants in our previous studies^3,14^ judged the softness of materials through haptic or visual exploration, whereas in the here reported Study 1, they only saw the written name of the materials. Therefore, their judgments were not dependent on direct visual or haptic information but instead on the activation of the corresponding material representation that entailed their knowledge of and experience with this material^11^. While we initially concerned that this might elicit a large variance in participants’ judgments,—because potentially each person’s experience with and exposure to a specific material is very idiosyncratic, leading to qualitatively quite different representations of the same material—however, our data suggest that participants were very consistent in their judgements (cf. also^11^). Perhaps this was because our participants (students in their 20 s) were exposed to a fairly similar set of materials and interactions. Using more diverse and less frequently used materials and a more diverse set of participants, might have led to different results.

### Representation of perceived softness activated by material names

We compared the representational structure of softness activated by words to the one activated by visual and haptic sensory information^3,14^. Similarities in the descriptive space obtained from PCAs between verbal, visual, and haptic conditions suggest that representations activated by words correspond to representations derived from sensory input : softness spaces derived from every condition shared the same three dimensions: *granularity*, *surface softness*, and *viscosity*. It is possible that the high correspondence in softness spaces between verbal, visual, and haptic conditions might arise from a shared representation that combines softness information from all modalities. Alternatively, it could be that there exist multiple, similarly organized softness maps that each represent a given modality. In this scenario, it could be that softness is primarily perceived and represented by touch. However, this representation, perhaps for reasons of efficiency, might be altered while associations, visual experiences and existing conceptual knowledge are formed. The differences we observed in the dimensional structure of PCAs between haptic and verbal/visual static conditions appear to lend support to the latter possibility. It could also be that visual, but not haptic sensations in memory are associated with a specific material (name), which explain the better correspondence of representations in verbal and visual static conditions. This however should also yield large differences in material identification performance between those conditions. Yet, the results of Study 2 show that material naming was only somewhat better in the visual than in the haptic condition, and that identification in both conditions was far from perfect. So, identifiability cannot offer an explanation for the lesser correspondence between representations derived from haptic and verbal/static visual conditions.

Compared to the haptic and dynamic visual conditions, visually (static) and verbally activated spaces contained less detail of the sensory experience, i.e., only three dimensions (i.e., surface softness, granularity, and viscosity), and interestingly they appear to exclude deformability. It could be that participants simply relied on the same knowledge, i.e., the same activated representational space, in static visual and verbal conditions, and that this common representation lacked some specific information. This could happen, for example, if an assessment of a specific material attribute has not been made before, e.g., judging the elasticity of hand cream. The same could also be true for deformability, which is a kinematic property. It can be estimated directly through haptic interactions, but also from dynamic shape and texture cues available in videos^1,34,46–48^ especially if hand movements while a material is explored are shown^14,35,49^. If we never had to judge attributes related to deformability, this information might simply be lacking from the common representation, that verbal and (static) visual input activate. To test this idea could be the subject of future experiments.

### Filling in softness related information

The associations that make up the structure of a representation allow us to 'fill in' information about a material that is not directly available from the stimulus. For instance, seeing a picture of a sponge lacks information about its deformability, unless someone applies pressure to it. Our conceptual knowledge of sponges, or actual episodic memories of interacting with a sponge might allow us to fill in this information even though it is not visually present. This requires, however, the mapping between different sources of information (visual, haptic, conceptual) to be well-defined (e.g., one-to-one). For instance, if visual gloss can indicate haptic slipperiness^12^, visual gloss values need to be mapped to haptic slipperiness values in the representation. At a coarse level, previous research seems to suggest that this could be the case. For example, Baumgartner et al.^30^ showed that major visual and haptic material dimensions are highly corresponding. At a more detailed level, the current and our previous study^14^ extended these results, by showing that similar dimensions make up the representational space of softness derived from haptic, verbal, static visual, and dynamic visual experiments.

We explicitly tested whether this overall structural similarity was sufficient to allow potential filling and showed, with a linear discriminant analysis that it is possible to directly predict softness dimensions between verbal, haptic and the two visual conditions on the basis of attribute ratings in each. However, classification performances with data from the haptic experiment was lower than that with data from visual and verbal conditions (Fig. 3). For instance, *surface softness* did not map well between haptic and visual/verbal conditions. The discrepancy between the haptic and visual condition might be explained by our daily exploration habits. In daily life, we usually explore fine structures (e.g., on textiles) not by looking but by touching them. In line with this, Rakhin and Onkar^50^ found strong positive correlation in perceived smoothness between haptic and visual exploration conditions of textiles when using close-up high-resolution images. However, this relationship was weaker when using more distant views of the textiles. It could be, that in our studies visual stimuli might lack the detail that would allow closer correspondence between visual and haptic representations. Despite these discrepancies, overall, classification results show that structural similarity provided sufficient information to predict material properties across modalities from the information available in each condition. But would it be also sufficient to directly predict mapping of specific materials between representations derived from verbal, visual and haptic experiments? The second classification analysis showed that this is indeed possible, and was especially successful for predictions between verbal and (static) visual conditions. Confusion errors for predictions of specific materials to and from the haptic condition (Fig. 4) followed the same patterns as those of the first classifier (Fig. 3), for example, the dimensions of *surface softness* and *viscosity* were confused in the first classification analysis and similarly hairy and viscous materials were confused in the second. Moreover, when a material was misclassified, it was usually confused with another material that had also high values on the Barlett scores of the same softness dimension. In essence, materials that mapped similarly onto the various softness dimensions were also easier confused with each other.

## Conclusion

Humans are consistent and reliable at judging softness on the basis of material name prompts. The estimated perceptual softness dimensions in the verbal condition are similar to those estimated from haptic and visual experiments and specifically, they share the dimensions of *granularity, viscosity,* and *surface softness*. However, our analyses also suggest that correspondence between verbally derived softness spaces and visually derived ones is higher. This was surprising, since softness is a mechanical quality that is best assessed through the haptic sense. We rule out that this better correspondence between verbally and visually derived spaces is an artifact of the ability to identify a material easier in the visual condition. Overall, our results show that the representation of soft materials entails both haptic and visual information that can be activated by merely reading the material name. This representation is as detailed as when activated through direct sensory experience. The richness of this representation likely explains how it is possible to fill in information about material attributes when the sensory information is insufficient.

## Methods

### Study 1

#### Participants

A total of 132 students from Giessen University took part in the online study (96 female, 36 male, *M*_*age*_: 21.6). All methods are ethically approved by Local Ethics Committee of Faculty 06 at Justus Liebig University Giessen and performed in accordance with Helsinki declaration^51^ except for pre-registration. Participant gave written informed consent before the experiments. All participants received either 8 €/h or course credits for their participation.

In the previously reported visual and haptic experiments^14^, 90 students participated: static visual condition: 20 females, 10 males; *M*_age_ = 23.4, age range: 20–31; dynamic visual condition: 21 females; age range: 20–33; *M*_age_ = 25.1; haptic condition: 21 females, 9 males; *M*_age_: 23.6, age range: 18–38. All participants were right-handed and reported no sensory, motor, or cutaneous impairments (haptic experiments), had or corrected-to-normal visual acuity and normal color vision^52^ (visual experiments).

#### Stimuli

##### Verbal experiment

Stimuli were the names of materials that we had used in earlier work^3,14^, presented to the participants as written words^3^. In short, the specific materials were chosen to score high on a given softness dimension (i.e., surface softness, granularity, viscosity, deformability, and roughness). The full list of materials and their corresponding dimensions were: deformability (sponge and playdough), viscosity (hand cream and hair gel), furriness (velvet, fur, and cotton balls), granularity (sand and salt), roughness (control dimension for haptics: sandpaper and felt), control (stress balls, cranberries, aluminum foil, linen, lentils, pebbles, paper balls, and wool). Also adjectives were the same as in previous work^3^ (to ensure comparability), and were selected to represent softness dimensions. The full list of adjectives and the corresponding dimensions were as follows: furriness (fluffy, hairy, soft, and velvety), viscosity (moist, sticky, and wobbly), granularity (granular, sandy, and powdery), deformability (hard, inflexible, and elastic), and roughness (rough and smooth). Note, that the roughness dimension has also been used as a control dimension in haptic experiments^3^ in order to test the validity of the experimental paradigm.

##### Previous haptic and visual experiments^3,14^

In the haptic condition participants freely explored 19 real materials, were as they watched close-up photographs (2049 × 1464 pixels) of the same materials in the visual static condition. Traces of hand explorations were left whenever possible (e.g., run through marks for salt) in order to increase the availability of shape cues in the photographs. Finally, in the dynamic visual condition, participants watched previously recorded manual explorations of real materials (6 s at 60 frames per second at a resolution of 1012 × 1080 pixels). In previous work we found that people adapt their hand movements to the material, the task, and the interaction between material and task^3^. Therefore, video stimuli were chosen randomly from a pool of videos (of three observers) that predominantly depict the most frequent one or two hand movements for a given material and task. The resulting video set consisted of 57 videos (19 materials × 3 sets). Details of the movies and still images can be found in Cavdan et al.^14^.

#### Design and procedure

##### Verbal experiment

An online experiment was conducted (using testable.org) to test how people judge softness of different materials based on material name prompts. In the beginning of the experiment participants received written instructions stating that they would be receiving a list of adjectives describing different material qualities. In each trial an adjective was presented on the participant’s screen for two seconds followed by a material name. Participants rated how much that adjective applies to the material on a five-point scale (1, does not apply; 5, strongly applies) (see Fig. 1). The order of adjectives and materials was randomized.

##### Previous visual & haptic experiment^3,14^

Figure 1 illustrates that the same procedure was basically also followed in the previous haptic and visual studies: in the visual static condition, participants pressed the space button after the adjective had been presented. Then an image appeared and stayed on the screen for 2 s; in the dynamic visual condition, after pressing the space button, a manual exploration video was presented. In the haptic experiment, after pressing the space button, a beep sound signaled the start of 4 s exploration duration and participants freely and actively explored the materials. During the exploration their vision was blocked with a curtain. Another beep signaled the end of exploration period and participant asked to disengage the exploration. Also here, the task of the participant was on every trial to rate the applicability of an adjective to the presented material.

### Study 2

#### Methods

##### Participants

52 volunteers from Giessen University participated in the experiment (31 female, 21 male, *M*_*age*_: 29.92). Participants in the haptic condition did not report any sensory, motor, or cutaneous impairments. Participants in the visual condition had normal or corrected-to-normal visual acuity based on self-reports. All methods were ethically approved by the Local Ethics Committee of Faculty 06 at the Justus Liebig University Giessen and performed in accordance with Helsinki declaration^51^ except for pre-registration. Participants provided a written informed consent prior to the experiment, and were compensated 6 € for their time.

##### Setup and stimuli

In Study 2, the same set of materials as in Study 1 were used. However, in the analysis we exclusively focus on the soft materials that loaded high in the common three softness dimension across verbal, visual, and haptic conditions.

In the haptic condition, active noise cancelling headphones (Sennheiser HD 4.50 BTNC) and earplugs were used to block any sounds that might be generated during material exploration. Hand movements of the participants were recorded with video camera. These were not analyzed for the current study. A horizontally rotatable armrest is used to keep the same exploration distance across participants as well as reducing the potential strain on the arm. A curtain was used to block the materials from participants' view. The experimenter sat behind the curtain and placed the stimuli on a plate (diameter 21.5 cm). Plates were fixed on the table for consistent presentation across participants and to eliminate the friction differences that might be caused by a moving plate. Materials that could be altered through exploration were replaced after each participant (e.g., hand cream, aluminum foil). Instructions were presented on a DELL UltraSharp monitor (resolution: 2560 × 1440) using custom scripts programmed in Matlab 2022a (MathWorks Inc., 2007) with Psychtoolbox routines^53^. A numpad and a keyboard were used for collecting responses.

In the visual condition, instructions and stimuli were presented on the same DELL UltraSharp monitor using custom scripts programmed in Matlab 2022a (MathWorks Inc., 2007) with Psychtoolbox routines^53^. A numpad and a keyboard were used for collecting responses. Stimuli were the same material images as in the previous static visual experiment^14^ and subtended an area of approximately 24° × 20°.

##### Design and procedure

Upon arrival in the laboratory, participants provided written informed consents and received the instructions about the experiment. In order to rule out the possibility of carry-over effects between visual and haptic conditions, a between-subjects design was used. Participants were randomly assigned to either the haptic or the visual condition. The overall procedure was similar to previous haptic and static visual experiments (used for analysis in Study 1), with the difference that they now first performed a softness dimension choice task, followed by a free material naming tasks. Prior to experimental trials (19) participants were allowed to practice in order to familiarize themselves with the procedure. For the practice trials a woodblock or its picture served as practice object.

In the haptic condition, participants were provided with earplugs and noise-cancelling headphones. On each trial, the experimenter placed a randomly selected material (e.g., play dough), and participant pressed a space-button when ready. This was followed by a beep sound which marked the start of a 4-s exploration period. Participant freely explored the material with their right hand and finished the exploration after another beep that signals the end of the exploration period. During the free exploration task, we recorded the hand movement of the participants from outside of their view for future hand movement analysis. By pressing a button on the numpad (1–5), participants selected which one of the following adjective group describes the material they just touched best: 1, fluffy/hairy/velvety; 2, wobbly/moist/sticky; 3, granular/sandy/powdery; 4, deformable/elastic/inflexible; 5, rough/smooth. Finally, they said out loud the name of the material they touched, and this response was recorded by the experimenter using a keyboard.

In the visual condition, participants sat in front of the computer where the instructions and the images were presented. They pressed space-button to indicate that they were ready. This was followed by a presentation of a material image (e.g., hair gel) for 2 s. Participants were asked to focus on the image while it was on screen. After the presentation of the image ended, participants performed the same two tasks as the haptic condition.

In total, participants performed 19 experimental trials (i.e., materials) excluding the practice. The haptic condition took approximately half an hour while the static visual condition lasted approximately 15 min.

## Supplementary Information

Supplementary Figures.

## Data Availability

The datasets used and/or analyzed during the current study available from the corresponding author on reasonable request.

## References

[1] Alley LM, Schmid AC, Doerschner K (2020). Expectations affect the perception of material properties. J. Vis..

[2] Callier T, Saal HP, Davis-Berg EC, Bensmaia SJ (2015). Kinematics of unconstrained tactile texture exploration. J. Neurophysiol..

[3] Cavdan M, Doerschner K, Drewing K (2021). Task and material properties interactively affect softness explorations along different dimensions. IEEE Trans. Haptics.

[4] Fei-Fei L, Fergus R, Perona P (2003). A Bayesian approach to unsupervised one-shot learning of object categories. Proc. IEEE Int. Conf. Comput. Vis..

[5] Fei-Fei L, Fergus R, Perona P (2006). One-shot learning of object categories. IEEE Trans. Pattern Anal. Mach. Intell..

[6] Lake BM, Salakhutdinov R, Tenenbaum JB (2015). Human-level concept learning through probabilistic program induction. Science (1979).

[7] Lake, B. M., Salakhutdinov, R. & Tenenbaum, J. B. One-shot learning by inverting a compositional causal process. In *Advances in Neural Information Processing Systems* (2013).

[8] Morgenstern Y, Schmidt F, Fleming RW (2019). One-shot categorization of novel object classes in humans. Vis. Res..

[9] Paulun VC, Gegenfurtner KR, Goodale MA, Fleming RW (2016). Effects of material properties and object orientation on precision grip kinematics. Exp. Brain Res..

[10] Tommasi, T. & Caputo, B. The more you know, the less you learn: From knowledge transfer to one-shot learning of object categories. In *British Machine Vision Conference, BMVC 2009—Proceedings* (2009). 10.5244/C.23.80.

[11] Fleming RW, Wiebel C, Gegenfurtner K (2013). Perceptual qualities and material classes. J. Vis..

[12] Adams WJ, Kerrigan IS, Graf EW (2016). Touch influences perceived gloss. Sci. Rep..

[13] Paulun VC, Kawabe T, Nishida S, Fleming RW (2015). Seeing liquids from static snapshots. Vis. Res..

[14] Cavdan M, Drewing K, Doerschner K (2021). The look and feel of soft are similar across different softness dimensions. J. Vis..

[15] Schmidt, F., Hebart, M. N., Schmid, A. C. & Fleming, R. W. Core dimensions of human material perception. PsyArXiv. 10.31234/osf.io/jz8ks (2022). 10.1073/pnas.2417202122 PMC11912425 40042912

[16] Gärdenfors P (2017). Semantic Knowledge, Domains of Meaning and Conceptual Spaces.

[17] Witzel C (2016). An easy way to show memory color effects. Iperception.

[18] Witzel C, Olkkonen M, Gegenfurtner KR (2016). Memory colours affect colour appearance. Behav. Brain Sci..

[19] Witzel C, Valkova H, Hansen T, Gegenfurtner KR (2011). Object knowledge modulates colour appearance. Iperception.

[20] Metzger A, Drewing K (2019). Memory influences haptic perception of softness. Sci. Rep..

[21] Caldiran O, Tan HZ, Basdogan C (2019). Visuo-haptic discrimination of viscoelastic materials. IEEE Trans. Haptics.

[22] Higashi K, Okamoto S, Yamada Y, Nagano H, Konyo M (2019). Hardness perception based on dynamic stiffness in tapping. Front. Psychol..

[23] Higashi, K., Okamoto, S., Yamada, Y., Nagano, H. & Konyo, M. Hardness perception by tapping: Effect of dynamic stiffness of objects. In *2017 IEEE World Haptics Conference, WHC 2017* (2017). 10.1109/WHC.2017.7989853.

[24] Lederman SJ, Klatzky RL (1987). Hand movements: A window into haptic object recognition. Cogn. Psychol..

[25] Klatzky RL, Lederman SJ, Metzger VA (1985). Identifying objects by touch: An ‘expert system’. Percept. Psychophys..

[26] Okamoto S, Nagano H, Yamada Y (2013). Psychophysical dimensions of tactile perception of textures. IEEE Trans. Haptics.

[27] Srinivasan MA, LaMotte RH (1995). Tactual discrimination of softness. J. Neurophysiol..

[28] Xu C, Wang Y, Hauser SC, Gerling GJ (2018). In the tactile discrimination of compliance, perceptual cues in addition to contact area are required. Proc. Hum. Factors Ergon. Soc..

[29] Xu C, Wang Y, Gerling GJ (2021). An elasticity-curvature illusion decouples cutaneous and proprioceptive cues in active exploration of soft objects. PLoS Comput. Biol..

[30] Baumgartner E, Wiebel CB, Gegenfurtner KR (2013). Visual and haptic representations of material properties. Multisens. Res..

[31] Bi, W. & Xiao, B. Perceptual constancy of mechanical properties of cloth under variation of external forces. In *Proceedings of the ACM Symposium on Applied Perception, SAP 2016* (2016). 10.1145/2931002.2931016.

[32] Drewing, K., Ramisch, A. & Bayer, F. Haptic, visual and visuo-haptic softness judgments for objects with deformable surfaces. In *Proceedings—3rd Joint EuroHaptics Conference and Symposium on Haptic Interfaces for Virtual Environment and Teleoperator Systems, World Haptics 2009* (2009). 10.1109/WHC.2009.4810828.

[33] Klatzky RL, Wu B (2014). Visual-haptic compliance perception. J. Vis..

[34] Schmid AC, Doerschner K (2018). Shatter and splatter: The contribution of mechanical and optical properties to the perception of soft and hard breaking materials. J. Vis..

[35] Wijntjes MWA, Xiao B, Volcic R (2019). Visual communication of how fabrics feel. J. Vis..

[36] Xiao B, Bi W, Jia X, Wei H, Adelson EH (2016). Can you see what you feel? Color and folding properties affect visual-tactile material discrimination of fabrics. J. Vis..

[37] Shinkareva SV, Malave VL, Mason RA, Mitchell TM, Just MA (2011). Commonality of neural representations of words and pictures. Neuroimage.

[38] Devereux BJ, Clarke A, Marouchos A, Tyler LK (2013). Representational similarity analysis reveals commonalities and differences in the semantic processing of words and objects. J. Neurosci..

[39] Vardar, Y., Wallraven, C. & Kuchenbecker, K. J. Fingertip interaction metrics correlate with visual and haptic perception of real surfaces. In *2019 IEEE World Haptics Conference, WHC 2019* (2019). 10.1109/WHC.2019.8816095.

[40] Efron B (1979). Bootstrap methods: Another look at the jackknife. Ann. Stat..

[41] Bates CJ, Yildirim I, Tenenbaum JB, Battaglia P (2019). Modeling human intuitions about liquid flow with particle-based simulation. PLoS Comput. Biol..

[42] Schmid AC, Doerschner K (2019). Representing stuff in the human brain. Curr. Opin. Behav. Sci..

[43] Dövencioǧlu DN, Üstün FS, Doerschner K, Drewing K (2022). Hand explorations are determined by the characteristics of the perceptual space of real-world materials from silk to sand. Sci. Rep..

[44] Bergmann Tiest WM, Vrijling ACL, Kappers AML (2013). Haptic discrimination and matching of viscosity. IEEE Trans. Haptics.

[45] Stilla R, Sathian K (2008). Selective visuo-haptic processing of shape and texture. Hum. Brain Mapp..

[46] Bouman, K. L., Xiao, B., Battaglia, P. & Freeman, W. T. Estimating the material properties of fabric from video. In *Proceedings of the IEEE International Conference on Computer Vision* (2013). 10.1109/ICCV.2013.455.

[47] Bi, W. & Xiao, B. Perceptual constancy of mechanical properties of cloth under variation of external forces. In *Proceedings of the ACM Symposium on Applied Perception, SAP 2016* 19–23 (2016). 10.1145/2931002.2931016.

[48] van Assen JJR, Barla P, Fleming RW (2018). Visual features in the perception of liquids. Curr. Biol..

[49] Cellini C, Kaim L, Drewing K (2013). Visual and haptic integration in the estimation of softness of deformable objects. Iperception.

[50] Rakhin, K. & Onkar, P. Predicting haptic perception of textile texture and analysis between smooth-rough preferences through images. In *Proceedings of Tools and Methods of Competitive Engineering* (2018).

[51] World Medical Association. World Medical Association Declaration of Helsinki: Ethical Principles for Medical Research Involving Human Subjects. *JAMA* **310**, 2191–2194. 10.1001/jama.2013.281053 (2013). 10.1001/jama.2013.281053 24141714

[52] Ishihara S (2004). Ishihara’s Tests for Color Deficiency.

[53] Kleiner M, Brainard D, Pelli D (2007). What’s new in Psychtoolbox-3?. Perception.

