# Supplementary material for: Assessing the representational structure of softness activated by words
Source: Sci Rep. 2023 Jun 2;13:8974. doi: 10.1038/s41598-023-35169-6 (PMC10238515; doi:10.1038/s41598-023-35169-6)
Supplement: Supplementary file 1 — Supplementary Figures. [file 41598_2023_35169_MOESM1_ESM.pdf]

Supplementary material

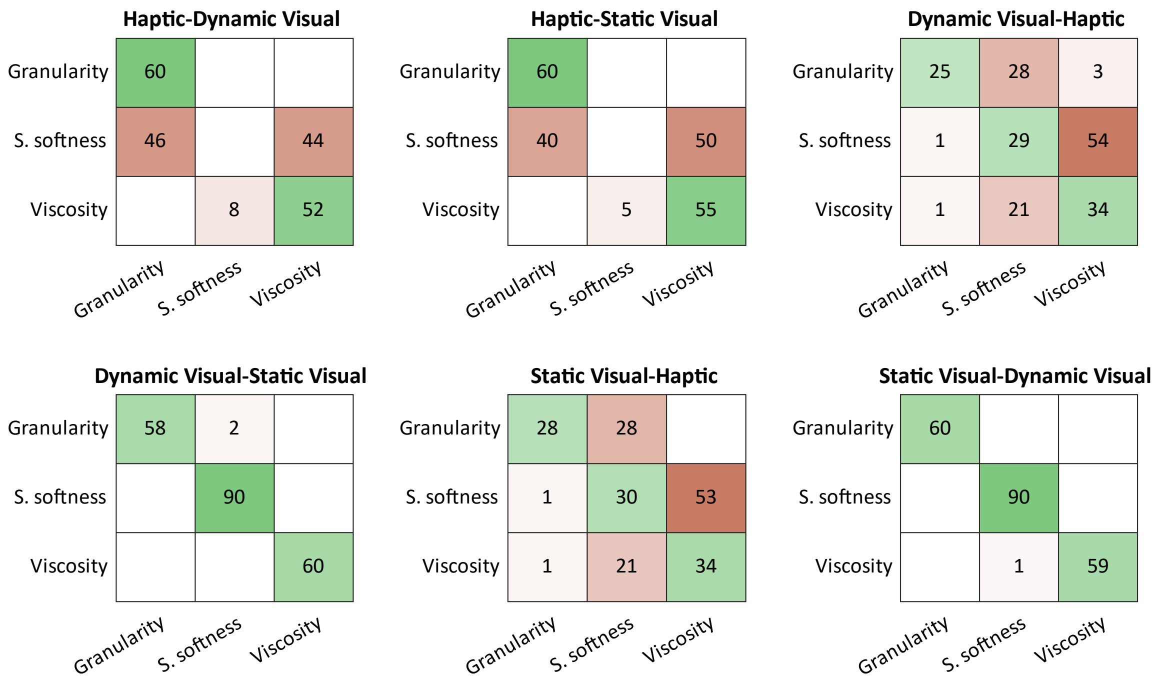

Figure 1. Confusion matrices showing the softness dimension classification performance of Static, Haptic, and Dynamic linear discriminant classifiers. Material classification performances as follows. **Haptic classifier**: Dynamic: 53.33%, Static: 54.76%, **Dynamic classifier**: Haptic: 43.56 %, Visual Static 99.05%. **Static classifier**: Haptic: 46.95%, Dynamic: 99.52%.

For completeness, here we report the remaining classification performances for the softness dimension classifiers. The classifiers trained on visual static and visual dynamic data were almost perfectly classify the visual condition. Whereas they frequently confused *granularity* with *surface softness* and *surface softness* with *viscosity* and vice versa when classifying haptic information. Despite of these systematic confusions, all classifiers were able to correctly classify *granularity* and *viscosity* of haptic information better than chance. However, only static visual and dynamic visual classifiers (i.e., classifiers trained on static or dynamic visual condition ratings) were able to classify haptic *viscosity above* chance.

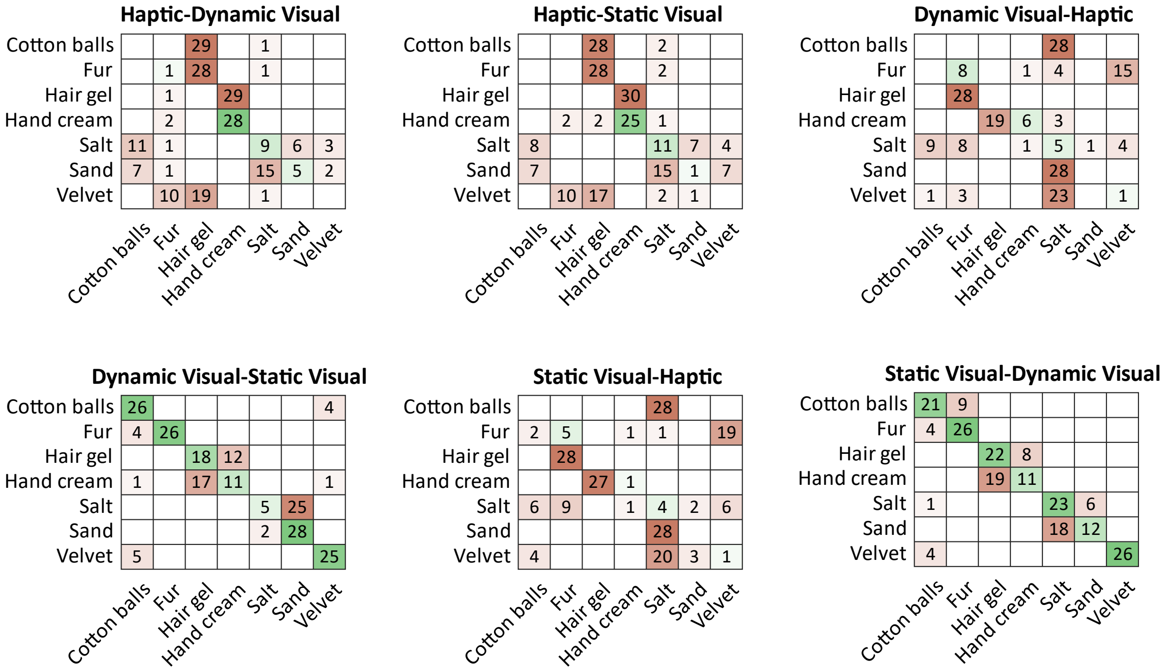

Figure 2. Confusion matrices showing the material classification performance of Static, Haptic, and Dynamic linear discriminant classifiers. Material classification performances as follows. **Haptic classifier**: Dynamic: 20.48%, Static: 17.62%, **Dynamic classifier**: Haptic: 10.2%, Static: 66.19%, **Static classifier**: Haptic: 5.61% , Dynamic: 67.14%. Numbers in each Similar to the main results, the material classifier trained on haptic data could significantly classify materials in visual conditions. However, again this negligible as most of the performance is attributed to *hand cream* classification. Visual classifiers had a reliable classification performance with errors mostly caused by within dimension confusions.

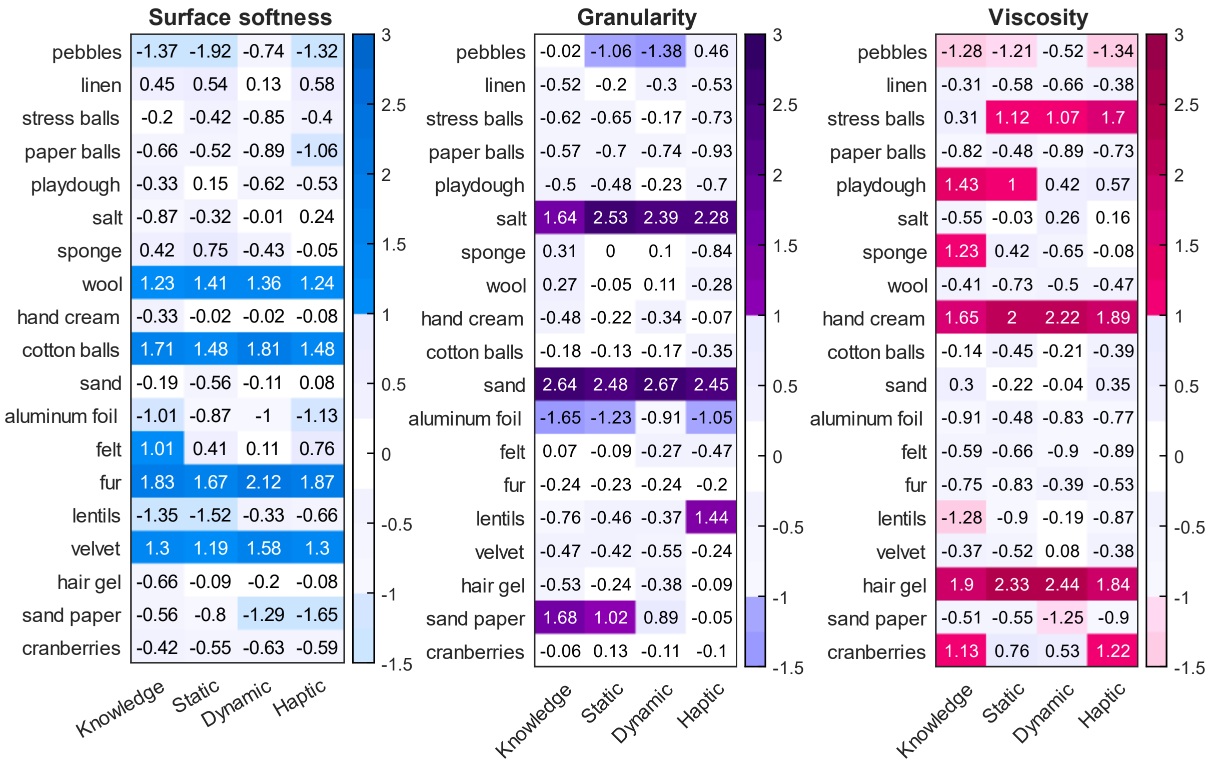

Figure 3. Rotated Bartlett scores of the materials in each extracted perceptual softness dimension: surface softness, granularity, and viscosity for verbal, static visual, dynamic visual, and haptic conditions, respectively. Darker colors indicate positive loadings while lighter colors indicate negative loadings.

During the classification, common positive loadings (+1 standard deviation) across all four conditions were used. Note that, although wool was loaded to surface softness dimension, it was not included in the classification as this material was initially selected as a control material in our previous studies (Cavdan, Doerschner, et al., 2021; Cavdan, Drewing, et al., 2021).

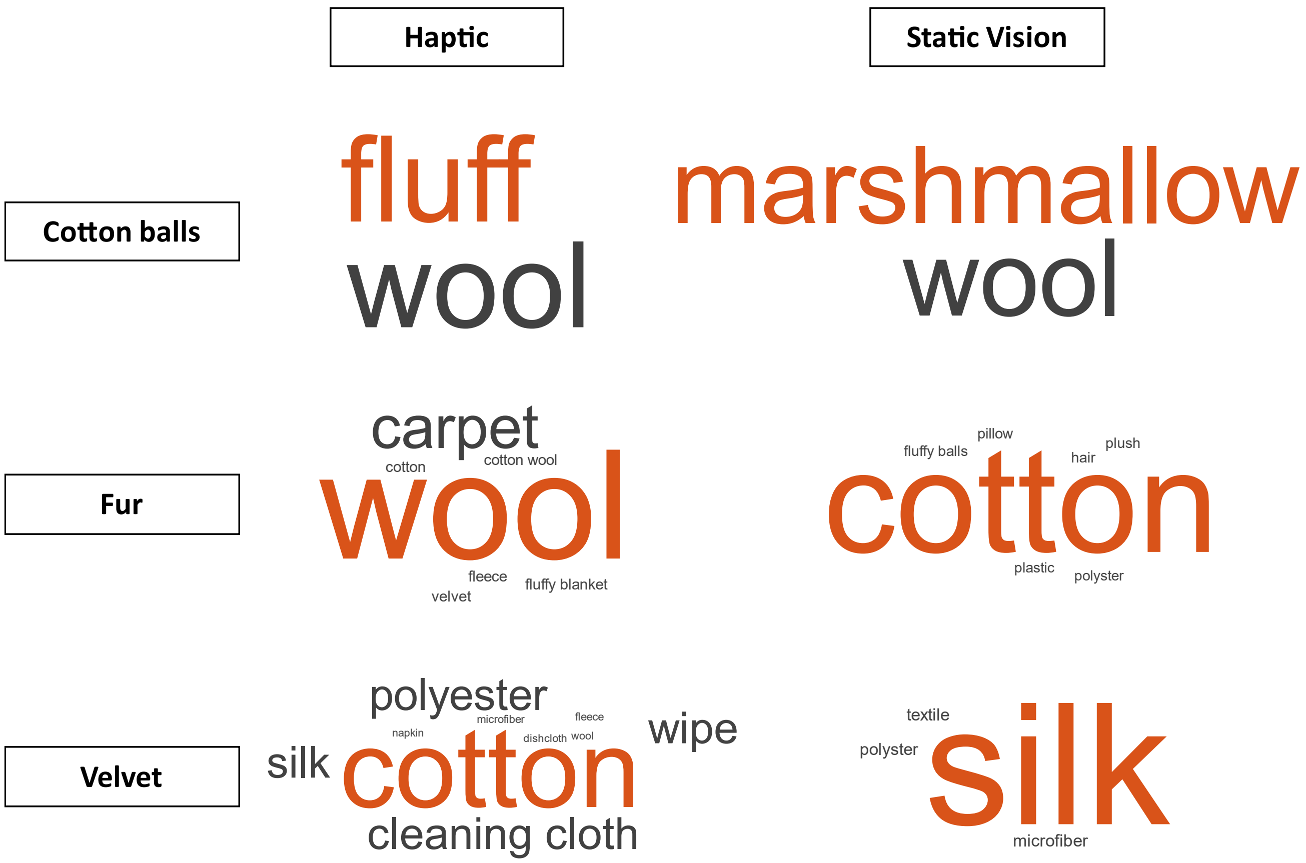

Figure 4. Identification errors for haptic (left column) and visual (right column) conditions for surface softness materials (top to bottom: cotton balls, fur, and velvet). Font size of the world clouds correspond to the error frequency. The most frequent confusion is highlighted in orange.

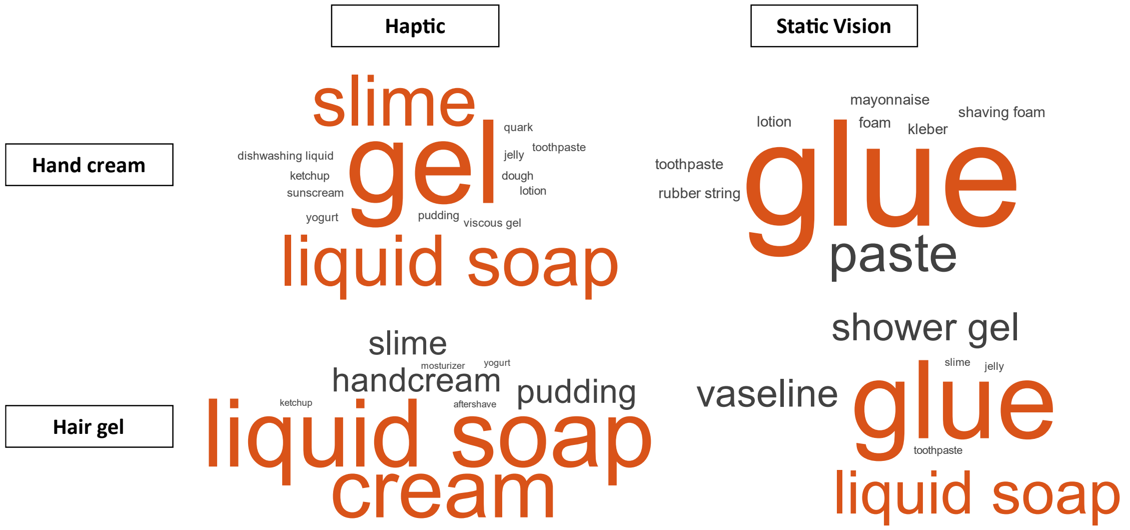

Figure 5. Identification errors for haptic (left column) and visual (right column) conditions for viscous materials (top to bottom: hand cream and hair gel). Font size of the world clouds correspond to the error frequency. The most frequent confusion is highlighted in orange.

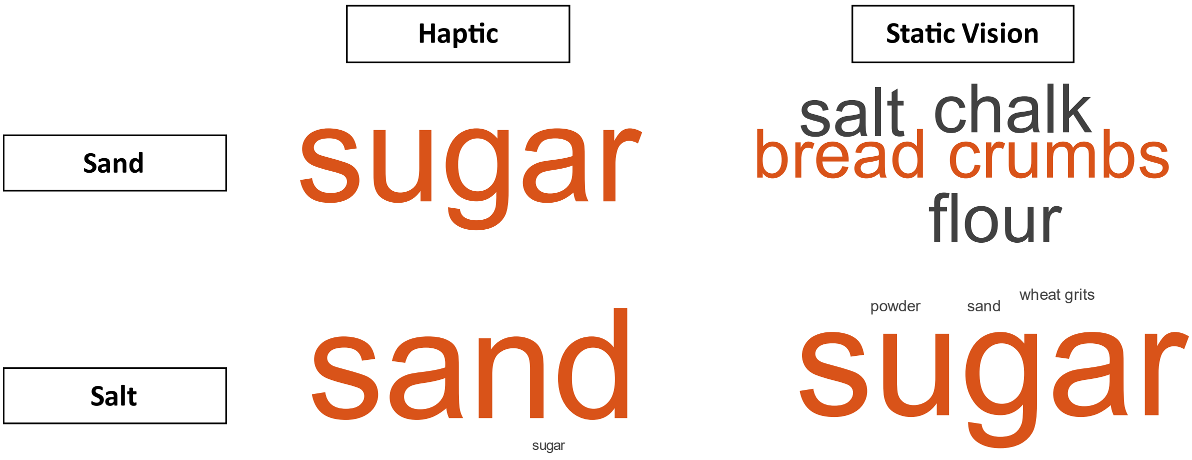

Figure 6. Identification errors in haptic (left column) and visual (right column) condition for granular materials (top to bottom: sand and salt). Font size of the world clouds correspond to the error frequency. The most frequent confusion is highlighted in orange.
